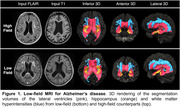# Portable, Low‐field MRI for Alzheimer’s Disease

**DOI:** 10.1002/alz.095305

**Published:** 2025-01-09

**Authors:** W. Taylor Kimberly

**Affiliations:** ^1^ Massachusetts General Hospital, Boston, MA USA

## Abstract

**Background:**

Low‐field magnetic resonance imaging (LF‐MRI) may facilitate point‐of‐care assessment of patients with Alzheimer’s Disease (AD) that can increase accessibility. However, image quality may be limited by lower signal‐to‐noise ratio (SNR). We developed a machine learning pipeline to quantify brain morphometry and white matter hyperintensities (WMH) using LF‐MRI, validated the volumes against conventional high‐field (HF) counterparts, and compared the volumes with aged controls.

**Methods:**

Patients with a diagnosis of mild cognitive impairment (MCI) or dementia due to AD (*n* = 45, age 73±8 years) were prospectively enrolled in the MGH Memory Division outpatient clinic. A separate cohort of controls (*n* = 23, age 64±8 years) were enrolled from the Yale Emergency Department and used for comparison. All patients underwent LF‐MRI acquisition on a 0.064T MRI which included T2 FLAIR and T1w sequences. We processed images through FreeSurfer‐based pipelines using *LF SynthSR* and *SynthSeg* for quantification of brain volumes and *WMH‐SynthSeg* to measure WMH burden. HF‐MRI (1.5‐3T) scans obtained within 8±12 months of LF‐MRI acquisition were used for comparison. Whole brain, hippocampal, and lateral ventricle volumes and WMH were quantified from HF and LF scans in patients and controls, normalized to intracranial volume (mean±SD).

**Results:**

Brain volumes quantified on LF‐ and HF‐MRI were correlated, including hippocampal (r = 0.82, 95% CI 0.72‐0.93), whole brain (r = 0.97, 95% CI 0.96‐1.0) and lateral ventricle (r = 0.98, 95% CI 0.96‐1.0) volumes (all p<0.0001). WMH burden was also correlated (r = 0.80, 95% CI 0.66‐0.91) between LF‐ and HF‐MRI scans. Compared to controls, hippocampal volumes were smaller among those with MCI or AD (2.3±0.2^−3^ versus 2.6±0.3^−3^, p = 0.0001). A similar reduction in whole brain volume was observed in MCI/AD patients (591.2±19.8^−3^ versus 621.3±20.5^−3^, p = 0.0001), which was accompanied by ventricular enlargement (18.6±7.4^−3^ *cf*. 13.1 ±7.3^−3^, p = 0.0007).

**Conclusion:**

Point‐of‐care LF‐MRI of patients with MCI or dementia due to AD is feasible and generates accurate brain volumes and WMH burden when combined with machine learning algorithms. Collectively, our findings demonstrate LF‐MRI’s potential in cost‐effective image evaluation for cognitive impairment that may reduce disparities in access. Our findings also highlight the future potential for routine longitudinal assessment, disease staging, and monitoring during anti‐amyloid treatment.